# Mobile supervised consumption services in Rural British Columbia: lessons learned

**DOI:** 10.1186/s12954-018-0273-3

**Published:** 2019-01-10

**Authors:** Silvina C. Mema, Gillian Frosst, Jessica Bridgeman, Hilary Drake, Corinne Dolman, Leslie Lappalainen, Trevor Corneil

**Affiliations:** 1 Interior Health Authority, Kelowna, British Columbia Canada; 2 School of Population and Public Health, University of British Columbia, Vancouver, British Columbia Canada; 3 Faculty of Medicine, University of British Columbia, Vancouver, Canada; 4 Department of Family Medicine, University of British Columbia, Vancouver, Canada

**Keywords:** Mobile supervised consumption services, Harm reduction, Overdose, Injection drug use

## Abstract

**Background:**

In 2016, a public health emergency was declared in British Columbia due to an unprecedented number of illicit drug overdose deaths. Injection drug use was implicated in approximately one third of overdose deaths. An innovative delivery model using mobile supervised consumption services (SCS) was piloted in a rural health authority in BC with the goals of preventing overdose deaths, reducing public drug use, and connecting clients to health services.

**Methods:**

Two mobile SCS created from retrofitted recreational vehicles were used to serve the populations of two mid-sized cities: Kelowna and Kamloops. Service utilization was tracked, and surveys and interviews were completed to capture clients’, service providers’, and community stakeholders’ attitudes towards the mobile SCS.

**Results:**

Over 90% of surveyed clients reported positive experiences in terms of access to services and physical safety of the mobile SCS. However, hours of operation met the needs of less than half of clients. Service providers were generally dissatisfied with the size of the space on the mobile SCS, noting constraints in the ability to respond to overdose events and meaningfully engage with clients in private conversations. Additional challenges included frequent operational interruptions as well as poor temperature control inside the mobile units. Winter weather conditions resulted in cancelled shifts and disrupted services. Among community members, there was variable support of the mobile SCS.

**Conclusions:**

Overall, the mobile SCS were a viable alternative to a permanent site but presented many challenges that undermined the continuity and quality of the service. A mobile site may be best suited to temporarily provide services while bridging towards a permanent location. A needs assessment should guide the stop locations, hours of operation, and scope of services provided. Finally, the importance of community engagement for successful implementation should not be overlooked.

In 2016, an unprecedented increase in the number of fatalities from illicit drug overdoses led to the declaration of a public health emergency in the province of British Columbia (BC), Canada. This surge in overdose deaths corresponded with an increased amount of synthetic fentanyl in the illicit drug supply. Fentanyl was found either alone or in combination with other illicit drugs in over 75% of overdose deaths investigated in BC in 2016 and 2017. The most common routes of consumption were injection (41%) and smoking (39%) followed by intranasal (29%) and oral (9%) [1].

Public health responses to the overdose emergency have primarily focused on large urban centres such as Vancouver, BC, while mid-sized and small communities are contending with the same emergency and fewer resources [2, 3]. To address the harms associated with illicit drug use, BC’s overdose emergency response has included expansion of medically supervised consumption services (SCS) across the province. Previously, SCS such as InSite and the Dr. Peter Centre were successfully implemented in Vancouver [4]. Internationally, there are over 90 SCS facilities throughout Western Europe, Australia, and Canada [5].

In Canada, clients accessing SCS bring their own pre-obtained illicit drugs into the facility, where they are provided with sterile injection equipment. This has been demonstrated to promote safer injecting practices and reduce needle sharing, as well as prevent transmission of blood-borne viruses (e.g. hepatitis C, HIV), other serious infections, and overdoses, all of which also place a financial burden on the health care system. SCS are effective entry points for clients to be referred to health and social services and can improve public safety by decreasing public injection and improper disposal of used syringes [5–8]. Despite the large body of evidence that supports SCS to mitigate some of the individual and population-level risks associated with injection drug use, implementation of SCS in smaller communities can be challenging [2, 3]. Major barriers include lack of concentration of drug use in one particular part of town, which requires providing small-scale services at multiple locations, and public perception that drug use is a “big town” problem.

Kelowna and Kamloops are two mid-sized cities in the Southern Interior of BC with municipal populations of 127,330 and 92,317, respectively [9]. Along with many other smaller cities in the province, these communities have been impacted by the overdose emergency. The number of overdose deaths in Kelowna increased from two deaths in 2008 to 76 in 2017, while the number of deaths in Kamloops climbed from seven to 38 deaths in the same time period [10]. In 2017, the overdose death rates in Kelowna and Kamloops were 60 and 41 per 100,000 population, respectively, both of which were substantially higher than the provincial overdose death rate of 30 per 100,000. Combined, these two communities represented about 8% of all overdose deaths in BC in 2017, yet they contained less than 5% of the total population.

An innovative delivery model using mobile SCS was piloted in Kelowna and Kamloops with the goals of preventing overdose deaths, reducing public drug use, and connecting clients to health services. This paper aims to share the results of an evaluation as well as lessons learned from the implementation of these services. The purpose of the evaluation was to systematically gather evidence to determine the strengths and limitations of such a model and determine the impacts that SCS may have had on these two communities.

## Methods

### Mobile supervised consumption services

**Fig. 1:**
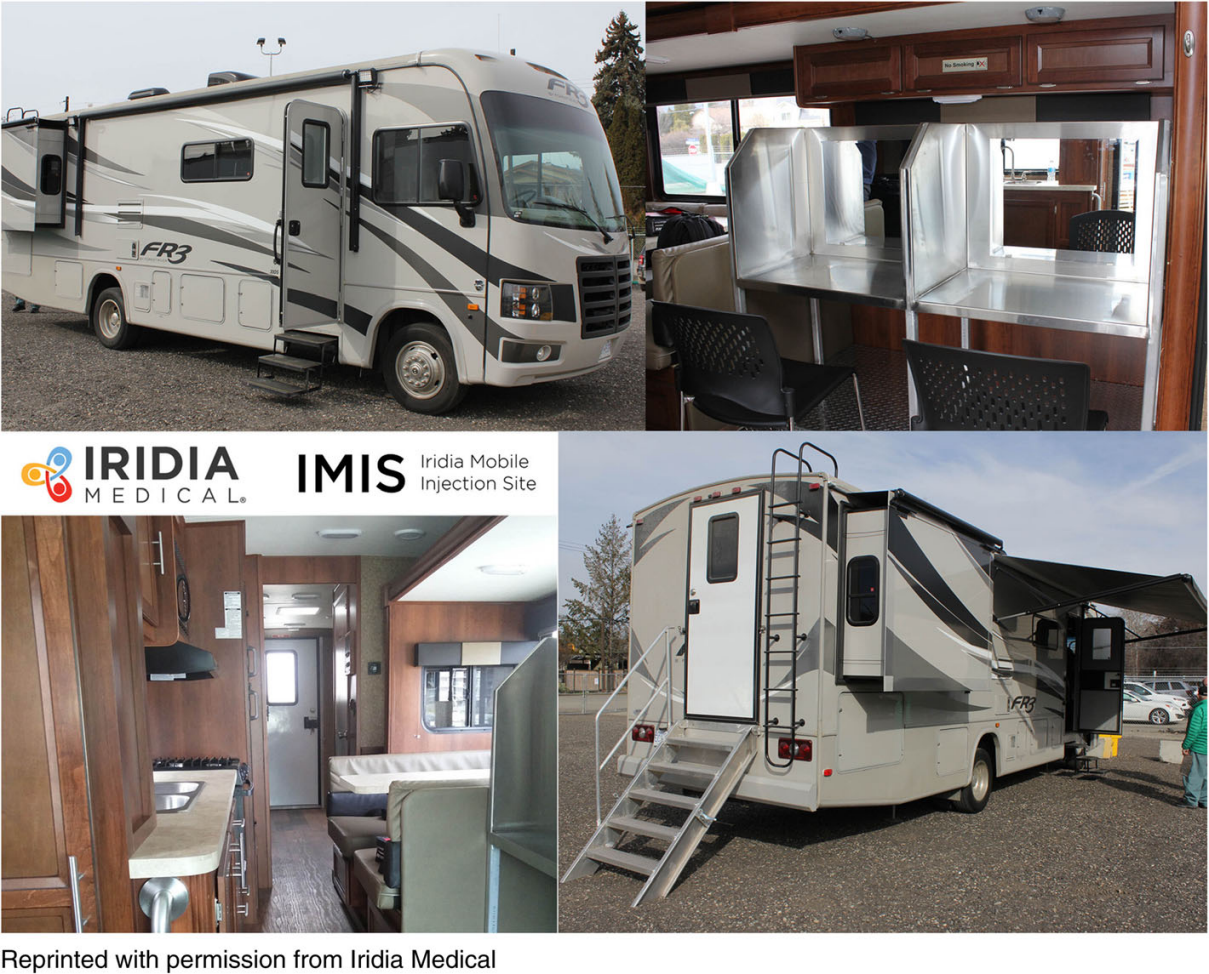
Visual of the outside and inside of the mobile supervised consumption services unit

Two mobile SCS units were created from retrofitted 35-ft recreational vehicles (RV). Each space includes two booths for clients to inject and additional areas for a waiting room and provision of medical services (Fig. 1). The only mode of consumption allowed in the mobile SCS is injection; other modes of consumption (e.g. smoking, intranasal) are not allowed. In addition to supervising injections, staff are available to monitor clients post-consumption, respond to overdoses if necessary, provide harm reduction supplies including naloxone kits, and provide nursing services (e.g. wound care) and referrals to medical and social services. At the time of the evaluation, the mobile SCS operated from Tuesday to Saturday and stopped at two different locations in each city. The mobile unit visited each location in four-hour shifts. In Kelowna, the stops were located in the downtown and Rutland neighborhoods. In Kamloops, the stops were located in the North and South Shore neighbourhoods.

The mobile units were initially opened as overdose prevention sites (OPS) following a BC ministerial order that allowed regional health authorities to provide overdose prevention services as necessary on an emergency basis [11]. OPS are designated spaces where people at risk of overdose can be monitored with personnel available to respond to overdoses when they occur. The mobile OPS opened in April and June of 2017 in Kelowna and Kamloops, respectively. In July 2017, an exemption under the *Controlled Drugs and Substances Act* [12] was obtained from Health Canada, which allowed the integration of supervised injection into the mobile services and transitioned the units from OPS to SCS.

### Evaluation process

An evaluation team was led by an independent evaluator. Members of the team included the mobile SCS operational managers, substance use and harm reduction clinicians, an epidemiologist, and a public health physician. The team developed a framework to establish the impact of the mobile SCS across five domains and included the perspectives of clients, service providers, and community stakeholders. For each domain, the level of success was rated as meeting criteria/good, approaching/ok, or unacceptable based on the responses to key evaluation questions (Table 1).

**Table 1:** Evaluation framework used to assess the impact of mobile supervised consumption services on clients, service providers, and community stakeholders

Evaluation domains	Evaluation questions
1. Implementation and programme delivery	1. Are the services being provided as intended at the mobile sites?
	2. To what extent are clients accessing services on the mobile units?
2. Client experience	3. How good is the client experience for those accessing services on the mobile unit?
3. Connection to services and health outcomes	4. Is the mobile service having a positive impact on client health outcomes? (e.g. overdose events, referrals, behaviours, primary care needs, successful linkage to other health services)
4. Provider experience	5. How positive is the provider experience?
5. Public experience and perceptions	6. To what extent are community stakeholders supportive of the mobile units?

### Quantitative data

Daily utilization of the mobile SCS was tracked by collecting information about client visits in a Microsoft Excel worksheet saved on a secure network. In an effort to keep access to the service as low barrier as possible, clients were only asked to provide a first name and/or alias upon registration and were not asked for their personal healthcare number or date of birth. Additional information collected included location of the service; date, time, and duration of the client visit; whether it was a first time or repeat visit; services and supplies provided (e.g. harm reduction supplies, naloxone, referrals); type(s) of substances consumed on the mobile unit (e.g. heroin, fentanyl, illicit stimulants); and associated overdose events. A referral, which intended to connect clients to health and community services, was defined as (1) speaking with the client about the service (education), (2) providing contact information for the service, and (3) verbal agreement on next steps for the client to access the service.

### Qualitative data

As part of the evaluation, client interviews were conducted on-site. Individuals who utilized the mobile SCS were approached by a staff member who provided information regarding the evaluation project and asked if they were interested in an interview with the lead evaluator. Participation was voluntary, and a five-dollar incentive was given to each client for participating. All interviews were conducted one-on-one by a single interviewer. Responses were recorded manually using a paper interview guide.

Feedback was sought from service providers and community stakeholders. Community *partners* included contracted agencies, municipal governments, and local police services, and community *members* included members of the public such as business owners, employees, and neighbourhood residents. A survey was developed for each of these groups and shared by e-mail. Community stakeholders were encouraged to forward the survey to others who may want to participate. See supporting documents for provider, community stakeholder surveys, and clients’ interview form.

### Data analysis

The qualitative data were reviewed and coded by the lead evaluator with support from a secondary coder. The primary coder read all qualitative entries twice to become familiar with the data and then created a coding system. At this point, an inductive approach using constant comparison was used. To ensure coding structure, a second coder reviewed the free text and codes and any differences were discussed. Codes were further developed and refined as needed.

### Ethics

This was not a research project and therefore did not require review by a Research Ethics Board. Instead, ethical risks were assessed using a framework designed for quality improvement and evaluation projects (A pRoject Ethics Community Consensus Initiative [ARECCI] Screening Tool) [13]. The data collection tools and associated methods were reviewed using the ARECCI Guidelines and Screening Tool and assigned a risk score of 7 (minimal risk category). As per the ARECCI recommendation, projects considered as minimal risk are not required to undergo a second opinion review.

## Results

### Implementation and programme delivery

From August to November 2017, there were 6105 visits to the Kelowna mobile SCS and 1865 visits to the Kamloops mobile SCS (Table 2). In Kelowna, the downtown location (in close proximity to an inner-city health clinic and a homeless shelter) accounted for 4579 (75%) of the visits and 589 (93%) of all injection events. In Kamloops, the majority (1100; 59%) of the visits to the mobile SCS occurred at the North Shore location. In contrast, 126 (56%) of injection events occurred at the South Shore location, where the mobile stopped in close proximity to an agency that provides harm reduction services and supplies. Among all injection events at the Kelowna and Kamloops mobile SCS, the durations of client visits were longer than 20 min in 411 (65%) and 140 (62%) of visits, respectively. In fact, 316 (50%) and 59 (26%) injection events exceeded 30 min in each community, respectively. A total of 626 naloxone kits were given out in Kelowna and 373 in Kamloops during the evaluation period.

**Table 2:** Overall mobile supervised consumption services utilization and injection events in Kelowna and Kamloops from August 1 to November 30, 2017

Month of visit	Total Kelowna visits	Total Kelowna injection events (%)	Total Kamloops visits	Total Kamloops injection events (%)
August	1393	169 (12.1)	555	76 (13.7)
September	1590	175 (11.0)	445	43 (9.7)
October	1533	122 (8.0)	472	45 (9.5)
November	1589	166 (10.5)	393	61 (15.5)
Total	6105	632 (10.4)	1865	225 (12.1)

Operational challenges were identified within weeks of implementation. The mobile units initially had two main entry/exit points for clients, one at the front and one at the back. Movement of the unit when access doors opened and as people walked inside the unit caused physical instability for clients injecting. The back stairs on the mobile unit were noted to be too steep, which posed a safety risk for staff and clients; the stairs were replaced. In an effort to improve the flow of clients through the unit, the back door became both the entry and exit point for clients accessing the services. This caused the rear section of the unit, the main area where clients received medical care, to become crowded as clients waited to access the injection booths that were located near the front of the unit. Temperature regulation inside the unit was also difficult with indoor temperatures becoming too hot in the summer and too cold in the winter due to the repeated opening and closing of the doors. Winterization of the mobile units required that all water be drained to prevent freezing, which resulted in handwashing sinks, taps, and toilets becoming unusable. Poor road conditions during ice and snow storms also created service disruption as the RVs were not safe to drive during these times. Furthermore, while parked, a third-party snow removal service was required to remove snow from the roof of the mobile units in order to operate them.

### Client experience

A total of 82 interviews were conducted among clients who accessed the mobile SCS (Kelowna *n* = 34; Kamloops *n* = 48). Of these, 56 (68%) were males, 59 (72%) reported having unstable housing, and 34 (41%) self-identified as aboriginal. Sixty percent of the clients interviewed were between 20 and 39 years of age. When using the service, 29 (35%) reported consuming substances on the mobile unit.

Overall, clients expressed a very positive experience with the service and the providers. Of the clients interviewed, 75 (91%) reported feeling physically safe accessing services on the mobile unit, being able to easily move around in the unit, and liking the mobile unit’s SCS locations. A total of 76 (93%) reported not feeling judged when accessing the service, and 80 (98%) reported no issues getting in/out of the mobile. However, only 34 (41%) clients reported that the hours of operation of the mobile SCS met their needs. Themes that emerged from the client interviews included friendly/inviting/welcoming staff, receiving education/advice from staff, assistance with referrals by staff, the mobile being a safe place in case of overdose, more responsible drug use, less needle sharing, and increased health-seeking behaviours and practices. Some clients expressed having a more positive outlook on life and/or life without drugs.

### Connection to services and health outcomes

Between August and November 2017, service providers in the mobile SCS responded and successfully reversed a total of 23 overdoses in Kelowna and seven overdoses in Kamloops. No overdose deaths occurred on the mobile service at either location. A total of 237 referrals to either health or social services occurred during the evaluation period across the two sites. The majority of these represented referrals to mental health and substance use treatment services. Table 3 shows a summary of the referrals from the mobile SCS in each community.

**Table 3:** Referrals initiated for mobile supervised consumption services clients in Kelowna and Kamloops From August 1 to November 30, 2017

Type of referral	Kelowna	Kamloops
Opioid agonist treatment	15	34
Withdrawal management	14	13
Shelter	10	19
Other mental health and substance use services	9	30
Residential services	6	15
Food	0	12
Others*	17	43
Total referrals	71	166
Total client visits	6105	1865
% referrals (total referrals/total clients seen × 100)	1.2	8.9

*Included financial support, specialized medical care, and other community/social services (e.g. grief, aboriginal)

### Provider experience

**Fig. 2:**
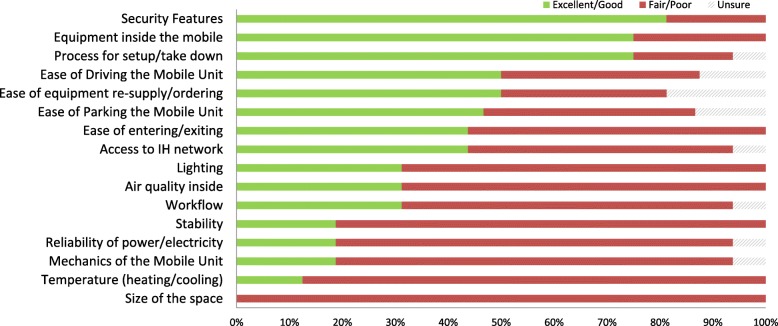
Provider ratings on aspects of the mobile units (*n* = 16)

The provider survey was emailed to full- and part-time staff (*n* = 21) working on the mobile units. Sixteen responses were received resulting in a response rate of 71%. There was an equal representation from providers across both communities. The survey asked the providers to rank a variety of aspects of the mobile units’ space and surroundings. Figure 2 provides a summary of the ratings by providers.

Survey responses from the providers mainly contained feedback about the physical space of the mobile units. Fourteen (87.5%) out of the 16 respondents reported that the limited space in the mobile impacted the ability to respond to an overdose event and the ability to have a private/confidential conversation. Safety-related themes that emerged from the qualitative coding of the survey centred on clients’ behaviours being unpredictable, aggressive, and compounded by the small space, as well as the risk of accidental injury through exposure to open sharps. More positively, 12 (75%) providers reported seeing health-promoting changes in clients. Examples of the comments provided are included in Table 4.

**Table 4:** Comments provided by service providers working in the mobile supervised consumption services

Ability to respond to an overdose	
“The physical space in the unit is absolutely unacceptable. It’s unsafe when a client overdoses and unsafe for staff and clients after they use.”	
“If two overdoses were to occur at once, limited space inside makes it difficult.”	
“We knew up front that the RV is a small space and that this would provide challenges when we are responding to a serious overdose. We have developed workflows and routines to make the best use of the space.”	
“I have not had an overdose while two people are using the site, however I do think it could pose some risks if it were to happen, due to limited space.”	
“Space is very awkward and small when having to respond to overdoses”	
Ability to have private/confidential conservations	
“...There are some aspects that I feel could be improved. I feel it isn’t always trauma informed and we can’t always provide confidential and optimal standard of care due to lack of space. We have a lot of requests for primary care especially in Rutland and we aren’t always able to provide the care needed that is much needed. At times participants seem to feel uncomfortable due to lack of space and don’t feel comfortable using because of all the people in the small space.”	
“The space is limited to accommodate only a small number of clients at a time, and the consumption booths are often full with clients waiting in cue to use the service. The space also does not provide adequate privacy for clients to share/discuss personal information.”	
“No space for confidential conversations either in person or via telephone due to only two areas with no sound barrier or place to meet with clients and accompanying supports.”	
“...it is a small space that doesn’t allow for much privacy for the individuals wanting to use the site. If a social worker wants to have a clinical conversation with a client it will always be within earshot of another person. If there are more than one client on the RV; or, another waiting to access for harm reduction supplies then it’s difficult to keep the momentum of a conversation moving forward. From a medical perspective there is little room for a nurse to carry out their duties as there is nowhere to examine a client. Clients have also mentioned how strange it is that two workers are so close to them while they are preparing to use.”	
General size	
“Very tight space to provide consumption services and try to do Intake info with clients.”	
“There is not a lot of space in the unit itself. It feels quite full when we have two people in the site using.”	
“Simply not big enough for number of clients accessing services and staff accessibility to equipment and also confidentiality; too narrow by bathroom to walk from back to front easily for more than one person at a time; safety and visibility for staff needing to support each other is an issue.”	

### Public experiences and perceptions

Community stakeholders were surveyed to determine their attitudes towards the mobile SCS. Partners included non-governmental organizations contracted by the health authority to deliver services to people who use drugs, municipal governments, and local police services. Of the 23 respondents in Kelowna, 13 represented local government, nine represented community agencies, and one was categorized as “other”. Of the 22 respondents from Kamloops, 17 represented the local police force, four represented local government, and one represented a community agency. Overall, the majority of community partners’ responses from Kelowna were supportive of the mobile SCS (60% strongly agree/agree, *n* = 14), while in Kamloops, the majority did not support the SCS (86% strongly disagree/disagree, *n* = 19) stating that visible drug consumption, drug litter, drug-related crime, and public nuisance events had worsened since the introduction of the service.

**Fig. 3:**
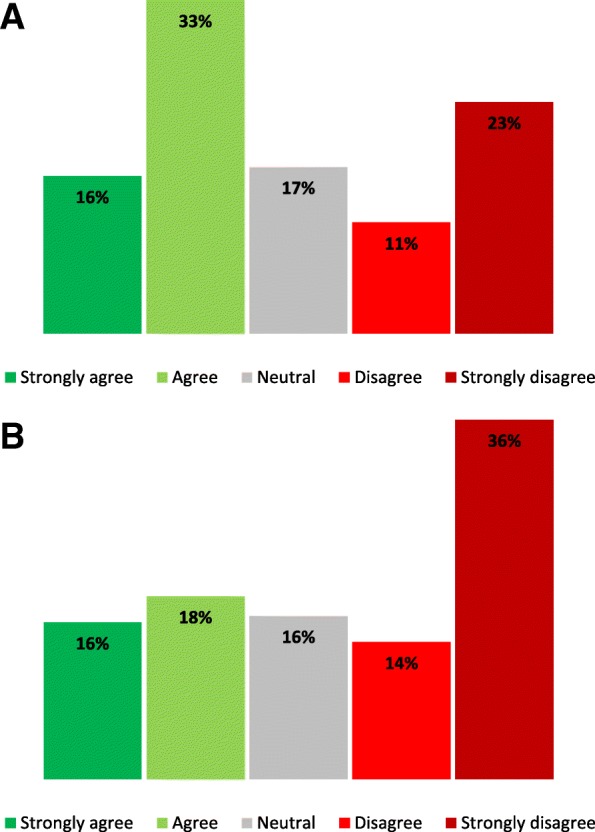
Community stakeholder survey responses to the question “In general, I am supportive of the idea of supervised consumption services” in Kelowna *n* = 112 (**a**) and Kamloops *n* = 153 (**b**)

Community members included business owners, employees, and residents in the neighbourhoods where the mobile SCS operated. A total of 112 and 153 community members replied to the survey in Kelowna and Kamloops, respectively. A higher proportion of respondents in Kelowna reported being supportive of the service compared to those in Kamloops (Fig. 3). These results were fairly consistent between the different locations within each city. Specific concerns noted in the survey were the perception of a general increase in drug activity, negative impact on public safety, and visible drug paraphernalia. Comments from community members are shown in Table 5.

**Table 5:** Comments by community members related to the mobile supervised consumption services

General positive comments	
“People’s lives have been saved”	
“As the problem of fentanyl lacing becomes worse and more people are affected by it, the more important supervised consumption sites become.”	
“I feel that there are more resources now in our community for people struggling with addictions, which is great to see….”	
“The challenges continue to grow. We increasingly need to support solutions not criticize the problem.”	
Negative comments (majority)	
“The service has attracted more drug addicted individuals in the area. The education portion does not seem to be working. There does not seem to be any available information / follow up on the drug use prevention side of things.”	
“Huge increase in amount of needles left in the streets and parks. Seems like there are more addicts in my area now, more trash, more human feces in public areas. I don’t feel we are helping these addicts at all, only helping them to destroy their lives further.”	
“Crime has gone up in this area, can no longer walk downtown after dark or on Sundays. Finding an increase needles in parks and sidewalks in the downtown core. Seen an increase in people high on drugs.”	
“Increased drug use around our building and parking areas. Our staff and clients feel less secure and we have had to increase our security (increased cost). Clients are commenting that feel less secure in the area.”	
“I have noticed a significant increase in drug consumption, loitering and litter near my workplace and parking space.”	
“My staff feel less secure in the area, I feel less secure in the area.”	

## Discussion

This paper summarizes the findings of the evaluation of an innovative mobile delivery model for the provision of SCS in two mid-sized communities in BC during an overdose public health emergency. Mobile SCS are a feasible option to reach people who inject drugs in smaller communities for a number of reasons. Firstly, mobile health services have been effective in increasing participation in primary and secondary prevention services such as immunizations [14], cancer screening [15], and harm reduction [16]. Secondly, mobile injection services have been successfully piloted in Copenhagen, Demark; Berlin, Germany; and Barcelona, Spain [17]. Thirdly, mobile SCS may enhance the ability to reach vulnerable populations at more than one location outside of large cities allowing flexibility to adapt to changes in the local drug scene. Lastly, a mobile delivery model can ameliorate neighbours’ and business owners’ concerns about SCS due to beliefs that these services promote drug use and crime, despite this concern not being supported by scientific literature [5, 18–21]. In other words, mobile SCS may be more palatable for the public and could pave the way for a future permanent site.

Overall, the mobile SCS were shown to provide a valuable service. However, the quality of the service was undermined by operational challenges such as service disruptions due to seasonal weather characteristics of the Southern Interior region of BC (i.e. hot summers and cold, snowy winters). Demand for the mobile SCS increased quickly after implementation, which pointed towards a possible underestimation of the need that only became apparent once the service was available. The demand was particularly apparent in Kelowna where the availability of alternative harm reduction services is otherwise limited. During the evaluation period, three times as many clients visited the Kelowna mobile SCS compared to the Kamloops services. The mobile units, each with only two injection booths, did not provide enough space to meet the needs of all clients who sought access to the services leading some to leave due to long wait times.

In addition to supervised injection, the mobile SCS also provided harm reduction supplies. This may have detracted from its primary purpose to offer a safer place for people to consume drugs and be monitored post-consumption. The proximity and capacity of an alternative source of harm reduction supplies is an important consideration when selecting a stop to ensure the SCS do not become overwhelmed by harm reduction supply and distribution. In Kamloops, one of the mobile SCS stops was located on the same property as a partner agency providing harm reduction and social services as well as other programmes. Co-location likely mitigated the demand for supplies from the mobile unit in this community, allowing staff more time for one-on-one interactions with clients.

Public perception of the mobile SCS was important for buy-in of community stakeholders directly impacted by the service. In both cities, community acceptance remained an issue after the implementation of the mobile SCS. Public perception was deeply influenced by personal values despite wide efforts to disseminate evidence of effectiveness. The underlying philosophy of harm reduction was relatively novel in both communities, and harm reduction supplies and services were placed under the spotlight like never before, fuelled by media stories showcasing a controversial angle.

It is important to note that homelessness and public safety were and continue to be important issues facing these two communities. The announcement of the overdose public health emergency in April of 2016, and the constant media coverage that followed over several months, increased the visibility of drug use as a social problem bringing harm reduction into mainstream services. The implementation of a widely contentious service coupled with the congregation of street entrenched individuals around the mobile units increased the visibility of the SCS and likely impacted community acceptance. Notwithstanding some negative feedback in the evaluation, some individuals were very appreciative to have the service in their community, particularly among those personally affected (e.g. individuals struggling with substance use, family and friends of people who use drugs, people who had previously lost a loved one to an overdose).

Strengths of this study include its focus on clients and service providers, as well as broader community feedback on the mobile SCS immediately following implementation. Among the limitations, the evaluation data included only four months of operations and may not fully represent experiences with the mobile SCS. In addition, the scope of the evaluation did not include a review of the overdose response from a public health and social determinants of health lens that may influence access to and experience with the mobile SCS. Finally, this study was conceived as an evaluation and lacks the scientific rigour of a research project.

In conclusion, this study demonstrates the feasibility of implementing mobile SCS in mid-sized cities during an overdose public health emergency that is largely driven by fentanyl contamination in the illicit drug market. In this context, mobile SCS is a viable service delivery model which may address particular communities’ needs. However, the local environment and operational challenges with a mobile unit may deem this service more useful as a temporary measure to bridge towards a fixed, permanent site. A needs assessment is recommended to guide the stop locations, hours of operation, and scope of services provided. The mobile nature of the service can be an advantage for public perception and acceptance while engaging the public and partners in a dialogue around substance use.

## Abbreviations

ARECCI: A pRoject Ethics Community Consensus Initiative
BC: British Columbia
RV: Recreational vehicle
SCS: Supervised consumption services
